# Measuring the Inducible, Replication-Competent HIV Reservoir Using an Ultra-Sensitive p24 Readout, the Digital ELISA Viral Outgrowth Assay

**DOI:** 10.3389/fimmu.2020.01971

**Published:** 2020-08-06

**Authors:** Erin L. Stuelke, Katherine S. James, Jennifer L. Kirchherr, Brigitte Allard, Caroline Baker, Joann D. Kuruc, Cindy L. Gay, David M. Margolis, Nancie M. Archin

**Affiliations:** ^1^University of North Carolina HIV Cure Center, UNC Institute for Global Health and Infectious Diseases, Chapel Hill, NC, United States; ^2^Department of Medicine, UNC Chapel Hill School of Medicine, Chapel Hill, NC, United States; ^3^Department of Microbiology and Immunology, UNC Chapel Hill School of Medicine, Chapel Hill, NC, United States; ^4^Department of Epidemiology, UNC Chapel Hill School of Public Health, The University of North Carolina at Chapel Hill, Chapel Hill, NC, United States

**Keywords:** QVOA, DEVO, HIV, outgrowth, IUPM

## Abstract

Quantifying the inducible HIV reservoir provides an estimate of the frequency of quiescent HIV-infected cells in humans as well as in animal models, and can help ascertain the efficacy of latency reversing agents (LRAs). The quantitative viral outgrowth assay (QVOA) is used to measure inducible, replication competent HIV and generate estimations of reservoir size. However, traditional QVOA is time and labor intensive and requires large amounts of lymphocytes. Given the importance of reproducible and accurate assessment of both reservoir size and LRA activity in cure strategies, efforts to streamline the QVOA are of high priority. We developed a modified QVOA, the Digital ELISA Viral Outgrowth or DEVO assay, with ultra-sensitive p24 readout, capable of femtogram detection of HIV p24 protein in contrast to the picogram limitations of traditional ELISA. For each DEVO assay, 8–12 × 10^6^ resting CD4 + T cells from aviremic, ART-treated HIV + participants are plated in limiting dilution and maximally stimulated with PHA, IL-2 and uninfected allogeneic irradiated PBMC. CD8-depleted PHA blasts from an uninfected donor or HIV-permissive cells (e.g., Molt4/CCR5) are added to the cultures and virus allowed to amplify for 8–12 days. HIV p24 from culture supernatant is measured at day 8 by Simoa (single molecule array, ultra-sensitive p24 assay) confirmed at day 12, and infectious units per million CD4 + T cells (IUPM) are calculated using the maximum likelihood method. In all DEVO assays performed, HIV p24 was detected in the supernatant of cultures as early as 8 days post stimulation. Importantly, DEVO IUPM values at day 8 were comparable or higher than traditional QVOA IUPM values obtained at day 15. Interestingly, DEVO IUPM values were similar with or without the addition of allogeneic CD8-depleted target PHA blasts or HIV permissive cells traditionally used to expand virus. The DEVO assay uses fewer resting CD4 + T cells and provides an assessment of reservoir size in less time than standard QVOA. This assay offers a new platform to quantify replication competent HIV during limited cell availability. Other potential applications include evaluating LRA activity, and measuring clearance of infected cells during latency clearance assays.

## Introduction

With an estimated 40 million people living with HIV (PLWH), and given the health, stigma, and financial burden associated with chronic HIV infection, eliminating the HIV pandemic remains a priority both from a public health and societal perspective. While successful antiretroviral therapy (ART) has significantly reduced the mortality and morbidity associated with HIV infection, the existence of long-lived viral reservoirs capable of reigniting fulminant infection in the absence of ART remains one of the major barriers toward achieving an HIV cure. With two documented functional cures, first the Berlin Patient (1) and more recently the London Patient (2), there is renewed hope and interest in the quest to eliminate persistent HIV infection. Modalities to target HIV persistence are being tested in the clinic. A large proportion of persistent HIV is defective and unable to replicate (3–5). Clinical interventions targeting the HIV reservoir would benefit greatly from assays that can rapidly and precisely quantitate the replication competent HIV reservoir in order to assess the efficacy of therapeutic interventions aimed at depleting the reservoir. Standard PCR-based assays offer a relatively rapid and sensitive method to quantitate persistent HIV infection. However, as most of these assays amplify one conserved genomic region, they do not distinguish between replication-competent and defective provirus (3–5). The recently reported Intact Proviral DNA Assay (IPDA) has the added advantage over standard PCR assays in that by using two sets of primer probes targeting an intact packaging signal (PS) and the Rev-responsive element within *Env*, it increases the probability of amplifying mostly intact proviral genome (6). Although relatively streamlined and amenable to high throughput, 30–40% of virus amplified by this method is likely to be defective, and sequence polymorphism may limit the ability of primers and probes to amplify intact provirus (6, 7). The QVOA is considered the gold standard to measure replication-competent, inducible provirus. The QVOA provides a minimal, but definitive estimate of the inducible HIV reservoir (8–10). However, this assay can be costly and labor intensive. Additionally, as latently infected CD4 + T cells are present at low frequency, large numbers of cells are often required to increase sensitivity. Furthermore for some participants, the QVOA may under-represent the true frequency of latent but replication-competent proviruses due in part to the presence of “non-induced” proviruses unresponsive to a single round of cell stimulation (4, 11). Despite its limitations, the QVOA remains the most reliable method to measure replication competent HIV (12). Thus several modifications of this assay have been made to improve throughput, sensitivity and increased its dynamic range [reviewed in Falcinelli et al. (13)]. We report here a modified QVOA, the Digital ELISA Viral Outgrowth or DEVO assay which takes advantage of the Simoa platform (Quanterix Inc., Billerica, MA, United States) (14, 15). The Simoa or single molecule array is an ultrasensitive, fully automated immune assay platform capable of femtogram detection of HIV p24 protein in contrast to the picogram limitations of traditional ELISA (15–17). During the DEVO assay 8–12 × 10^6^ purified resting CD4 + T cells from aviremic, ART-treated HIV + participants are PHA stimulated in limiting dilution in a 96 well-format and HIV p24 measured by Simoa.

To reduce non-specific signal, we use an optimized Simoa p24 protocol (16) in our assay. We found that virus can be expanded using either the CD4 T cell input alone (i.e., addition of exogenous donor cells is not necessary), PHA blasts from an uninfected donor, or HIV permissive cell lines such as the MOLT4/CCR5. Furthermore, with the DEVO assay, we obtained IUPM comparable or higher than the traditional QVOA at an earlier time point, thus reducing the overall length of the assay (18). While there have been other reports using the Simoa as a p24 readout for other QVOA modifications (19–21), to our knowledge, this is the first study describing a specific Simoa HIV outgrowth assay that has been meticulously and carefully evaluated for demonstrable accuracy and reproducibility.

## Results

### Participant Characteristics

To develop the DEVO assay we used resting CD4 + T cells isolated from 12 PLWH, stably suppressed (<50 copies of HIV-1 RNA/ml on ART). Participants were 75% male and 25% female, had a mean age of 43.5 years with an average CD4 count of 740 cells/μl, and on ART for an average of 7.3 years, with a mean duration of suppression of 4.9 years (Supplementary Table S1). Leukapheresis or whole blood samples were obtained from participants through an ongoing longitudinal collection protocol approved by the University of North Carolina (UNC) biomedical institutional review board. All samples were collected in accordance with UNC guidelines and all participants provided informed consent prior to sample donation.

### Simoa Detects HIV *gag* p24 Earlier Than Standard p24 ELISA During QVOA

Our primary goal for developing the DEVO assay was to investigate whether or not using ultra-sensitive p24 measurements by Simoa would shorten the duration of standard QVOA by detecting HIV p24 positive wells earlier than traditional ELISA methods. To that end, we harvested supernatant from a standard QVOA assay on days 8, 15, and 19 post-stimulation and measured HIV p24 by both Simoa (day 8) and standard HIV *gag* p24 ELISA (days 8, 15, and 19). Seventy three percent of the wells were p24 positive by Simoa at day 8 compared to 13% of the wells by standard ELISA (Table 1). Importantly, 95% of the wells slated to become positive at day 15 during the traditional QVOA assay were already positive at day 8 by Simoa and the IUPM at day 8 were comparable to the day 15 traditional QVOA IUPM (Table 1).

**TABLE 1:** HIV antigen is detected earlier using Simoa compared to standard ELISA in the traditional QVOA^Ψ^.

	Number of HIV p24 positive wells/total wells cultured		
	
	Day 8		Day 15

Resting CD4 + T cells (×10^6^)	Simoa	Standard ELISA	Standard ELISA
2.5	18/18	2/18	18/18
0.5	4/6	2/6	5/6
0.1	0/6	0/6	0/6
IUPM	1.927	0.088	2.49

*^Ψ^ Representative QVOA experiment.*

We next performed a pilot experiment to determine whether the sensitivity of the Simoa would be maintained when fewer cells are used for the viral outgrowth assay. We stimulated approximately 8 million resting CD4 + T cells in limiting dilution as described under methods. Culture wells were then targeted with PHA blasts to amplify virus. We subsequently assessed p24 production from culture supernatant at day 8 post-stimulation. We observed that detection of p24 positive wells by Simoa was more sensitive with 10 positive wells detected, as compared to 3 wells by standard ELISA (Supplementary Figure S1).

### Determination of the Lower Limit of Quantitation of the DEVO Assay

**FIGURE 1:**
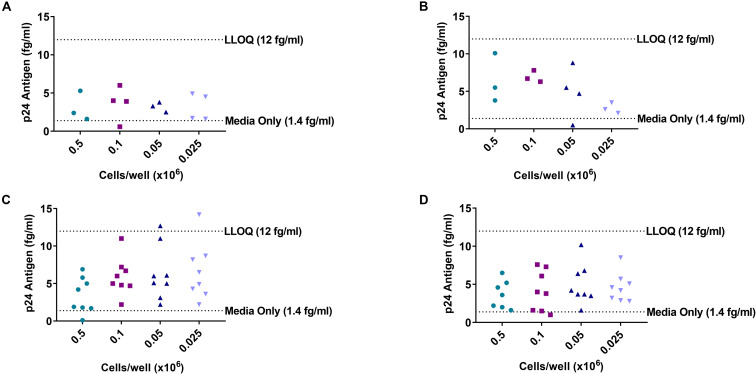
Limiting dilution culture of uninfected donor PBMC reveals background noise of Simoa. PBMC from an uninfected donor were stimulated in limiting dilution with PHA/IL-2 and irradiated PBMC or treated with low amount of IL-2 only. Cultures were either targeted with PHA blasts or no targets were added. Supernatant were harvested at day 8 and HIV p24 assayed by Simoa. **(A)** Low IL-2 treated/no targets added. **(B)** Low IL-2 treated/cultures targeted with PHA blasts. **(C)** PHA stimulated/no targets added. **(D)** PHA stimulated/cultures targeted with PHA blasts. Each dot represents a well at the indicated cell dilution. LLOQ, lower limit of quantitation.

During the development of the DEVO assay, we initially used the lower limit of quantitation (LLOQ) generated from the Simoa assay standard curve, which is approximately 12 fg/ml and 3 times the median absolute deviation (MAD) as the cut off value from which to assign a well as positive. Additionally, to reduce non-specific noise in the assay, we employed an optimized Simoa p24 protocol developed by our colleagues at Merck Pharmaceuticals (16). However, despite these careful approaches, we observed that in some cases, wells that were low-positive for HIV p24 would become negative if cultured for additional days, suggesting further modification of the DEVO assay was necessary to capture true replication competent-HIV and avoid recording poorly adapted virus that are incapable of spreading in culture. To better understand the level of background noise in the assay, we performed a mock viral outgrowth assay using PBMC isolated from an uninfected donor. Approximately 2–6 million PBMC were maximally stimulated in limiting dilution with PHA/IL-2/irradiated PBMC or with survival amount of IL-2 (10–20 U/ml) and cultured over 8 days. Cultures were targeted with PHA-blasts or no targets were added. Supernatant from cultures were harvested on day 8 and assayed by Simoa, using the optimized HIV p24 protocol referenced. Although false p24 signal was detectable in most of the wells, the values were below the limit of quantitation of the assay except for two wells in the PHA-stimulated, no target added culture conditions where values above the LLOQ were registered (Figure 1).

**FIGURE 2:**
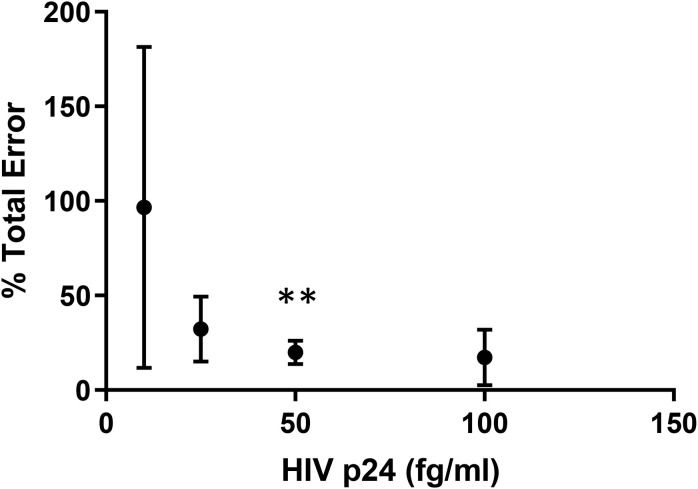
The lower limit of quantitation for the DEVO assay. Freshly isolated resting CD4 + T cells from an uninfected donor were stimulated with PHA and irradiated PBMC, then co-cultured with PHA blasts over the course of 19 days. On three separate, but not consecutive days, supernatant from day 8 and 12 were spiked with HIV p24 protein at different dilutions and assayed by Simoa. Bias, total error and % total error was calculated from replicate well values using the following formula: Bias = Average−*ActualValue*;*TotalError* = |Bias| + 2×*StandardDeviation*;%*TotalError* = *TotalErrorActualValue*×100. The concentration with <20% total error was determined to be 49.21fg/ml**. This value was used as the DEVO lower limit of quantitation.

To eliminate the influence of low-level false p24 positive signal in the assay, we next employed the methods standardized by The Clinical and Laboratory Standards Institute (CLSI) to determine a new limit of quantitation based on total error of the assay (22). Resting CD4 + T cells were isolated from a normal donor and stimulated with PHA/IL-2/irradiated PBMC for 24 h. Cultures were washed to remove the PHA and targeted with PHA-blasts from an uninfected donor twice over the course of 19 days. On three separate days, supernatant harvested on days 8 and 12 post-stimulation were spiked with different concentrations of HIV p24 (Quanterix, Inc.) and assayed by Simoa. Values obtained were used to calculate assay bias, total error and percent total error as described under methods. The concentration at which there was <20% of total error was found to be approximately 50 fg/ml and this value was used as the DEVO assay LLOQ onward (Figure 2). In addition, for added rigor and to eliminate recording cryptic p24 signal from wells containing only defective provirus, only wells exhibiting sustained or increase in p24 production over the 2 days of the assay are scored as positive (Supplementary Tables S2, S3).

### Comparison of the DEVO Assay to the Traditional QVOA

**FIGURE 3:**
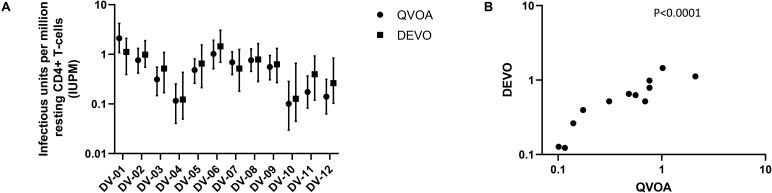
Comparison of IUPM values obtained in the DEVO assay at day 8 post-stimulation versus IUPM values obtained from the QVOA at day 15 post-stimulation. **(A)** The DEVO assay generated IUPM values that were either comparable or in some cases higher than values obtained with the traditional QVOA. However, overall, there was no significant difference in IUPM values between the two assays. *P* = 0.1294, Wilcoxon matched-pairs signed rank test. Twelve independent, paired assays from 12 distinct participants is shown. IUPM + 95% CI (upper and lower limits) is shown. **(B)** Correlation of the DEVO assay at day 8 post-stimulation with the QVOA at day 15 post-stimulation. Spearman *r* = 0.9282.

**FIGURE 4:**
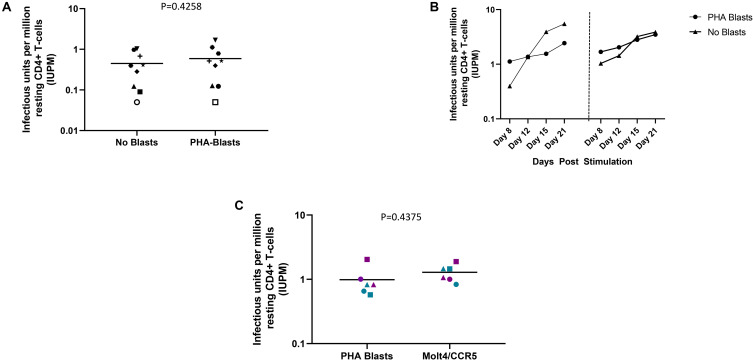
DEVO assay outcome using either no blasts, or PHA blasts or the HIV permissive cell line, Molt4/CCR5 to expand virus. **(A)** Increase in IUPM values over time in cultures receiving either PHA blasts or no exogenous targets (no blasts). Two independent assays using cells from two participants are shown (left and right panel, DV-01 and DV-02). **(B)** Similar IUPM values are obtained in the DEVO assay whether or not exogenous targets to expand virus are used. Each symbol represents an independent assay performed with cells from distinct participants. **(C)** The HIV permissible cell line Molt4/CCR5 expands virus as well as PHA blasts in the DEVO assay. Results from independent assays performed with cells from three different participants (DV-01, DV-05 and DV-06) at day 8 (green) and day 12 (purple) are shown. For each experiment, cultures either received Molt4/CCR5 or PHA blasts to expand virus. Each symbol represents a participant. Open symbols indicate values below the limit of detection. Wilcoxon matched-pairs signed rank test was used to compare statistical differences.

Using the optimizations defined above, we next compared the performance of the DEVO assay to the traditional QVOA in measuring the replication competent HIV reservoir of stably suppressed, ART-treated PLWH. Both assays were set-up in parallel using resting CD4 + T cells isolated from HIV + ART-suppressed donors. The QVOA was performed as described elsewhere (12, 23) using 34–50 million resting CD4 + T cells that are maximally stimulated with PHA/IL2/irradiated PBMC in limiting dilution. PHA-blasts from an uninfected CCR5 high donor are added to the cultures twice to expand virus and supernatant harvested on days 15 and 19 and assayed for HIV p24 by ELISA. For the DEVO assay 8–12 million resting CD4 + T cells are stimulated in limiting dilution as done for QVOA. Wells were either targeted with PHA blasts or in corresponding experiments received no blasts in order to assess whether virus can be expanded without the addition of exogenous cells. Culture supernatant were initially harvested on days 8, 12, 15, and 19 and p24 measured by Simoa. For both assays, the maximum likelihood method (24, 25) was used to calculate IUPM values. At day 8 post-stimulation, the DEVO assay generated IUPM values that were comparable to or in some assays higher than QVOA IUPM obtained at day 15 post-stimulation (Figure 3A and Supplementary Figure S2). However, the overall difference in IUPM between the DEVO at day 8 and the QVOA at day 15 were not statistically significant. Importantly, IUPM values for the two assays highly correlated (Figure 3B). Interestingly, similar IUPM values were obtained in the DEVO assay whether or not exogenous cells were added to expand virus, suggesting that the assay can be performed without the addition of target cells (Figure 4A). Additional days in culture beyond day 12 post-stimulation increased the number of positive wells in some, but not all assays (Figure 4B; not shown). This is similar to the standard QVOA where in most assays, all wells containing HIV outgrowth are p24 positive by day 15 with very few or no additional wells turning positive with longer culture. As one of the goals of the DEVO was to reduce the length of the QVOA assay, and given that by day 8, IUPM values were comparable or higher than the traditional QVOA assay, day 8 p24 assessment with a day 12 confirmation were subsequently selected as the assay endpoint (Supplementary Tables S2, S3).

The use of HIV permissive cell lines such as Molt4/CCR5 instead of PHA-blasts to amplify virus outgrowth in co-culture assays could diminish the cost associated with PHA-blast production and eliminate donor-to-donor variability in longitudinal studies (26). We therefore compared the ability of Molt4/CCR5 cells to expand virus in the DEVO assay as compared to PHA blasts. We observed no significant difference in IUPM values whether Molt4/CCR5 or PHA blasts were used in the assay (Figure 4C).

### Longitudinal Variation of the DEVO Assay

**FIGURE 5:**
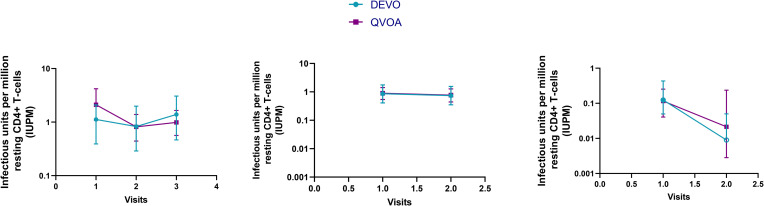
The DEVO assay tracked similarly overtime as the traditional QVOA. The DEVO assay and QVOA were performed with resting CD4 + T cells collected at multiple time points from 3 participants, DV-01 (first panel), DV-09 (second panel), and DV-04 (third panel). All assays were performed using cells from the same leukapheresis donation, except for VV-09 where the DEVO was performed using cells from whole blood donated within 3 months of leukapheresis donations used for the QVOA. The time elapsed between visits 1 and 2, 1 and 3, and 2 and 3 for DV-01 is 7.9, 14.1, and 6.2 months respectively; for DV-09 the time elapsed between visit 1 and 2 is 17.9 months (DEVO) and 14.9 months (QVOA); For DV-04, the time between the first and second donation is 15. 9 months. IUPM + 95% CI (upper and lower limits) is shown.

Longitudinal reservoir measurements are important metrics to evaluate the efficacy of therapeutic interventions and therefore depend on reliable and reproducible assays for accurate assessment. We previously reported on the longitudinal reproducibility of the traditional QVOA (12) and therefore, we compared the reproducibility of the DEVO assay with the QVOA. In two participants, the DEVO assay was run in parallel with the QVOA using resting CD4 + T cells collected longitudinally, spanning 6–14 months. In one participant, we compared the DEVO assay performed using whole blood resting CD4 T cells collected longitudinally (17.5 months between donations), with the QVOA performed using leukapheresis-derived resting CD4 T cells acquired within 3 months of the blood cells (15.2 months between donations). We observed that the DEVO assay tracked with the QVOA within a given participant and across participants over time (Figure 5).

**FIGURE 6:**
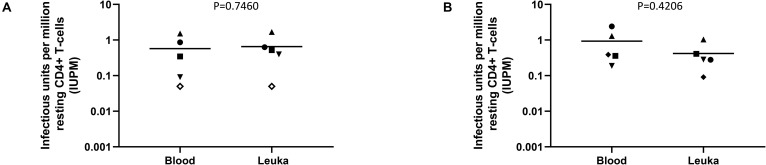
Comparison of the DEVO assay using resting CD4 + T cells isolated from whole blood vs leukapheresis product (Leuka). **(A)** Targets (either PHA blasts or MOLT4/CCR5) added to expand virus. **(B)** No targets added. Whole blood was collected 6–17 months from leukapheresis donation time. Each symbol represents an independent assay performed using cells from 4 distinct participants (DV-02, DV-03, DV-04, DV-09 and DV-11). For each experiment, cultures either received exogenous targets to expand virus or no targets. Open symbols indicate values below the limit of detection. The Mann–Whitney *U* test was used to compare statistical differences.

The ability to perform the DEVO assay using whole blood-derived instead of leukapheresis-derived resting CD4 + T cells would be beneficial for several reasons. It could reduce the costs associated with obtaining leukapheresis product and the time a participant spends in the clinic. Removing the need for leukapheresis procedures would also allow for the inclusion of additional time points to measure the replication competent reservoir during clinical trials. We therefore compared the performance of the DEVO assay on resting CD4 + T cells isolated from peripheral blood versus cells donated at leukapheresis in three different donors. We observed no significant difference in IUPMs obtained from the DEVO assay performed with the two different sources of resting CD4 T cells suggesting that the DEVO assay could be incorporated as an important tool in clinical trials seeking to deplete the HIV reservoir (Figure 6).

## Discussion

Interventions to deplete the HIV reservoir are being explored in the clinic and would greatly benefit from endpoint assays that can rapidly and reliably measure the replication competent HIV reservoir. While PCR assays to measure DNA and RNA are high-throughput, rapid and relatively streamlined, they do not distinguish between defective proviruses and replication competent virus (3–5). The newly described IPDA offers a significant advantage over traditional PCR methods targeting only a single genomic region of the virus as the IPDA uses primer/probe sets simultaneously targeting multiple conserved regions of the HIV genome to more accurately detect intact provirus. However, the IPDA still over-estimates the frequency of true intact proviruses (6, 7). Near full-length or full genome sequencing may provide better assessment of intact proviruses than standard DNA PCR, however, the inefficiency associated with long distance PCR for DNA sequencing, and the extreme costs and labor involved makes them difficult to apply on a large scale [reviewed in Falcinelli et al. (13)]. The QVOA, though labor intensive, provides a minimal estimate of the frequency of latent HIV infection, and is still the gold standard for measuring true replication competent HIV. We report here a validated, modified version of the QVOA which incorporates a digital p24 ELISA as a readout for sensitive and accelerated detection of HIV. We showed that the DEVO assay can be performed in less time and with fewer cells than the traditional QVOA. Importantly, IUPM values obtained from the DEVO assay were comparable or in some cases higher than the values from the traditional QVOA. Finally, we also show that the assay varies minimally longitudinally and can be performed using resting CD4 + T cells isolated from either whole blood or leukapheresis product. An optimized HIV p24 protocol is used in the DEVO assay to improve sensitivity (16). Additionally, to improve accurate measurement of replication competent virus, we meticulously defined a limit of quantitation for the assay and included an additional day of culture to confirm positive wells. While others have reported using the Simoa to quantitate the inducible reservoir, to our knowledge this the first report with detailed description of the steps taken to validate the assay and demonstrate longitudinal reproducibility and accuracy.

Given the importance of the QVOA in assessing replication competent HIV, it is no surprise that efforts have been devoted to streamlining the assay (13). Increasing sensitivity to detect viral outgrowth while minimizing cell input has been a common goal in the field. Other laboratories have also reported modifications of the assay to use fewer than 10 million cells while maximizing the ability to record the frequency of latently infected cells (19, 27, 28). One of the challenges of all viral outgrowth assays is the phenomenon of non-induced, replication competent virus (4, 11). Capturing cell free RNA from the supernatant of outgrowth wells using magnetic beads before assaying for HIV gag by PCR, was shown to increase sensitivity to detect inducible HIV (19, 28). However, while RNA measurements may provide more sensitivity, the detection of RNA may not represent a replication-competent viral particle. Further, false positive detection because of PCR contaminants could be a disadvantage. In a more recent report, effector memory T cells were found to contain a higher frequency of inducible HIV, leading to the suggestion that the effector memory state was overall more conducive to HIV latency reversal than other T cell differentiation states (29). This lead to the development of a modified QVOA termed the dQVOA, during which resting CD4 + T cells are first differentiated into effector memory T cells using a cocktail of cytokines before the stimulation and viral outgrowth steps (27). The differentiation step resulted in significantly higher frequencies of reactivated HIV compared to the traditional QVOA (27). Whether or not including this differentiation-to-effector-memory step in the DEVO assay would further increase the frequency of virus detected remains to be determined.

There are some limitations to our assay. Because we are interrogating fewer cells, the confidence interval around our point estimate (IUPM) is wide, especially when the frequency of latently infected cells is low. Thus in stably suppressed PLWH with extremely small inducible reservoirs, or if someday anti-latency interventions are able to significantly deplete the reservoir to very low levels, the applicability of the DEVO assay as described, in such situations will be limited as large numbers of cells will have to be interrogated to make an accurate assessment of the frequency of replication competent HIV. In such cases, either a modified DEVO assay or standard QVOA using large numbers of cells might be preferred. As an alternative, murine viral outgrowth models (mVOA or hmVOA) may provide an attractive, *in vivo* approach to record difficult to detect replication competent virus [(30, 31) reviewed in Schmitt and Akkina (32)]. Another limitation of our assay relates to selecting day 8 as the assay end point. As mentioned previously, in some assays, additional days of culture produce additional p24 positive wells. Thus wells that were p24 negative at day 8 may become positive at a later day but would not be considered positive, and result in a lower IUPM estimate than if the assay was extended to a later timepoint. However, there are obvious costs to extending times in culture, and a negative result (no positive cultures at the end of an assay) simply defines a limit of detection under the conditions employed. Increased sensitivity, at the burden of increased cost, may be obtained by extending time in culture.

In summary, the DEVO assay represents an advance to the available validated toolkit to measure replication competent HIV. The DEVO assay offers a new platform to quantify replication competent HIV for a variety of applications, such as measuring the frequency of infection in situations where the number of cells available may be limited, evaluating LRA activity, and measuring clearance of infected cells following the addition of autologous immune effectors.

## Materials and Methods

### Cell Culture

MOLT4/CCR5 cells were acquired from the NIH AIDS Reagent Bank. Cells were maintained in culture in RPMI supplemented with 1% penicillin/streptomycin (Gibco, ThermoFisher), 10% FBS (Gibco, ThermoFisher) and 1 mg/mL G418 until use.

PHA-blasts were prepared from PBMC obtained from selected HIV seronegative donors screened for adequate CCR5 expression (33). PBMC were CD8-depleted and maintained in culture in IMDM supplemented with 1% penicillin/streptomycin, 10% FBS and 20 U/ml IL-2. Cells were stimulated for 2–3 days with 2 μg/ml PHA prior to usage.

### Isolation of Resting CD4 + T Cells

HIV+ participants underwent continuous flow leukapheresis to obtain large amount of white blood cells or 150 ml of whole blood was obtained by venipuncture at a different time point from the leukapheresis from selected participants. PBMCs were isolated by Ficoll-gradient. Resting CD4 + T cells were isolated from PBMC by negative selection as previously described (34). Resting CD4 + T cells were maintained in culture for 1–2 days in the presence of ARV, without IL-2 prior to performing outgrowth assays.

Buffy coats from HIV seronegative donors were obtained from the New York Blood Center (New York, NY, United States) and PBMC isolated by Ficoll-gradient. Resting CD4 + T cells were isolated using the EasySep^TM^ Human Resting CD4 + T Cell Isolation Kit (StemCell Technologies, Vancouver, BC, United States). Cells were cultured overnight in media containing 20 U/ml IL-2 prior to assay set-up.

### Traditional QVOA

The QVOA assay was performed as previously described (12, 23). Briefly, 34–50 million resting CD4 + T cells were plated in replicate limiting dilutions of 2.5 million (12–18 cultures), 0.5 million (6 cultures) and 0.1 million (6 cultures) cells per well, activated with PHA (Remel, ThermoFisher) and a fivefold excess of allogeneic irradiated PBMCs from a seronegative donor, and 60 U/ml IL-2 for 24 h. Cultures were washed and co-cultivated with CD8-depleted PHA-blasts. Culture supernatants were harvested on days 15 and 19 and assayed for virus production by p24 antigen capture ELISA (ABL, Rockville, MD, United States). Cultures were scored as positive if p24 was detected at day 15 and was increased in concentration at day 19. A maximum likelihood method was used to estimate the frequency of resting cell infection, reported as infectious units per million CD4 + T cells (24, 25). Unless otherwise indicated, IUPM from day 15 post-stimulation of the QVOA is reported.

### DEVO Assay

Resting CD4 + T cells were plated in limiting dilution of a combination of 0.5 × 10^6^, 0.25 × 10^6^, 0.1 × 10^6^, 0.05 × 10^6^ and 0.025 × 10^6^ cells per well at 12 replicates each. Cells were stimulated with 2 μg/mL PHA, 60 U/mL Interleukin-2 (IL-2), and irradiated PBMCs from an HIV-seronegative donor. After 24 h, the cells were washed to remove the PHA, and MOLT-CCR5 or PHA-blasts were added at 0.05 × 10^6^ or 0.2 × 10^6^ per well respectively to amplify outgrowth of the virus. For experiments testing whether virus outgrowth would occur without the addition of exogenous feeder cells, no targets were added to the wells. Fresh media was added to cultures every 3–4 days. On day 8, the wells were split and targeted with another round of MOLT-CCR5 or PHA blasts. Supernatants were harvested on days 8, 12, 15, and 19 or just days 8 and 12. An optimized protocol was used to quantify HIV p24 antigen in culture supernatant by the Simoa HD-1 Analyzer (Quanterix, Billerica, MA, United States). Only wells exhibiting sustained or increase in p24 expression over the multiple days of harvest were scored as positive. A maximum likelihood method was used to estimate the frequency of resting cell infection, reported as infectious units per million CD4 + T cells (24, 25). Unless otherwise indicated, all DEVO assays were performed with the addition of exogenous targets (PHA blasts or Molt4/CCR5) to amplify virus and IUPM from day 8 post-stimulation is reported.

### Determination of DEVO LLOQ

Sero-negative donor resting CD4 + T cells were cultured in triplicate at 1 × 10^6^ cells/ml in cIMDM with 0.2 × 10^6^ irradiated normal donor PBMCs/ml, 3 μg/ml PHA and 100 units/ml IL-2. After 1 day, cells were washed one time to remove PHA. Cells were then co-cultured with target cells with wells receiving 0.4 × 10^6^ normal donor PHA-stimulated, CD8-depleted PBMCs per million resting cells. On day 5, a half of media was removed and replaced with an equivalent volume of media containing IL-2. On day 8, supernatant was collected, aliquoted and stored at −80°C. An equivalent volume of media containing IL-2 and the appropriate number of targets cells were added back to each culture. Half volume of supernatant was collected from each well on days 8,12, 15, and 19, aliquoted and stored at −80°C for later processing.

On three separate, but not consecutive days, Using Calibrator I from Quanterix Simoa HIV p24 kit, day 8 and day 12 supernatants were spiked in triplicate at 100, 50, 25, 10, and 0 fg/ml of p24 and assayed on the Simoa on. For each concentration, supernatant day and assay day, the average and standard deviation of the three replicates were used to determine the bias, total error and% total error using the following formula: Bias = Average − Actual Value; Total Error = | < *cps*:*it* > *Bias* < /*cps*:*it* > | + 2 × Standard Deviation; % Total Error = Total Error Actual Value × 100 (22). Percent total error versus concentration was plotted and fit using a 4-pl curve fit. From this fit, the concentration at which there is <20% total error was interpolated and found to be approximately 50 fg/ml. This value is used as the DEVO assay lower limit of quantitation.

### Statistical Analyses

All statistical analyses were performed using GraphPad Prism version 8.0.0. Wilcoxon matched-pairs signed rank or Mann-Whitney *U* Test were used as appropriated to compare differences between groups. For IUPM below the limit of detection, resting cell infection was estimated assuming that 1 culture at the highest input cells was positive, and one-half of this value was used for the statistical analyses (12). Correlations between the DEVO and QVOA were assessed using the Spearman correlation coefficient. A *p* value of less or equal to 0.05 was considered to be significant in all analyses performed.

## Data Availability Statement

All datasets generated for this study are included in the article/Supplementary Material.

## Ethics Statement

Samples were obtained from participants through an ongoing longitudinal collection protocol approved by the University of North Carolina (UNC) biomedical institutional review board. All samples were collected in accordance with UNC guidelines and all participants provided written informed consent prior to sample donation.

## Author Contributions

NA and ES conceptually designed the study. ES, KJ, JLK, and BA performed the experiments. NA, ES, and KJ performed the data analysis. NA wrote the manuscript. JDK, CB, DM, and CG provided clinical coordination and support. All authors edited and approved the final version.

## Conflict of Interest

The authors declare that the research was conducted in the absence of any commercial or financial relationships that could be construed as a potential conflict of interest.
