# Supplementary material for: Measuring the Inducible, Replication-Competent HIV Reservoir Using an Ultra-Sensitive p24 Readout, the Digital ELISA Viral Outgrowth Assay
Source: Front Immunol. 2020 Aug 6;11:1971. doi: 10.3389/fimmu.2020.01971 (PMC7423995; doi:10.3389/fimmu.2020.01971)
Supplement: Supplementary file 1 [file Data_Sheet_1.PDF]

# Measuring the inducible, replication-competent HIV reservoir using an ultra-sensitive p24 readout, the digital ELISA viral outgrowth (DEVO) assay.

Stuelke EL[1], James KS[1], Kirchherr JL[1], Allard B[1], Baker C[1], Kuruc JD[1,2], Gay CL[1,2], Margolis DM[1, 2, 3, 4] and Archin NM[1,2*]

[1]University of North Carolina HIV Cure Center, UNC Institute of Global Health and Infectious Diseases, [2]Departments of Medicine and [3]Microbiology and Immunology, UNC Chapel Hill School of Medicine, [4]Department of Epidemiology, UNC Chapel Hill School of Public Health, University of North Carolina at Chapel Hill, Chapel Hill, North Carolina.

*Correspondence should be addressed to: Nancie M. Archin PhD
nancie_archin@med.unc.edu

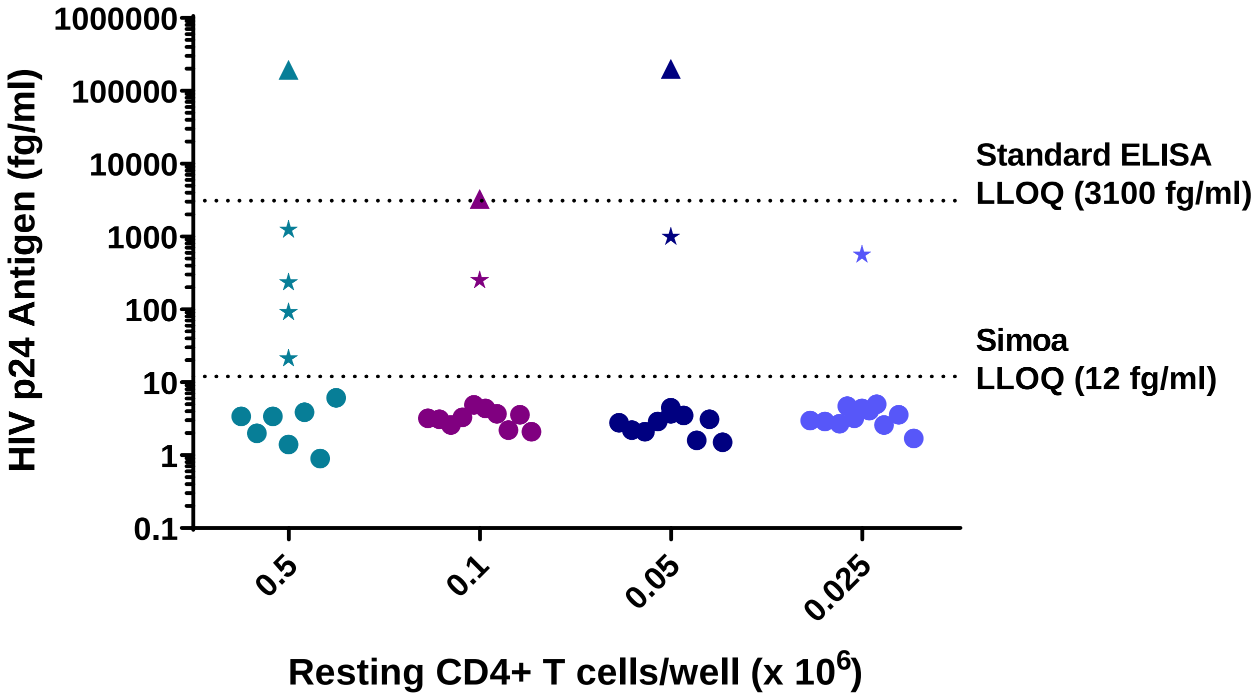


**Figure S1.** Ultra-sensitive p24 detection by Simoa versus standard ELISA in a viral outgrowth assay at day 8 post-stimulation. Each dot represents a well at the indicated cell dilution. Stars indicate p24 positive wells detected by Simoa only. Triangles indicate wells detected by both Simoa and standard p24 ELISA. LLOQ, lower limit of quantitation.

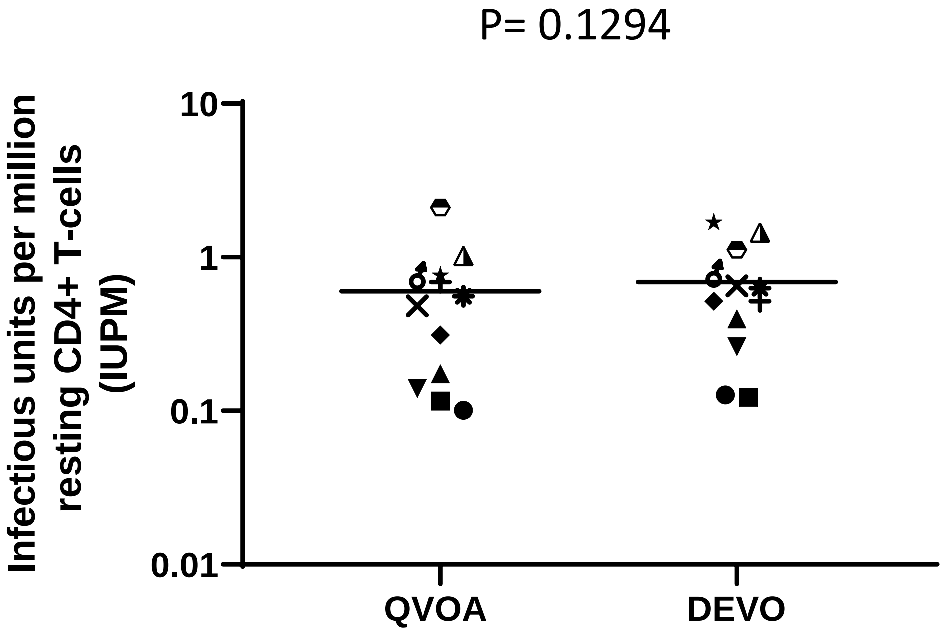


**Figure S2.** Comparison of IUPM values obtained in the DEVO assay at day 8 post-stimulation versus IUPM values obtained from the QVOA at day 15 post-stimulation. Wilcoxon matched-pairs signed rank test was used to compare differences.

**Table S1**. Clinical characteristics of HIV+ donors$^{\Psi}$

| Donor ID | Race | Sex | Age | CD4 Count | Years on ART | Years Suppressed* | ART Regimen | Treatment Status |
|---|---|---|---|---|---|---|---|---|
| DV-01 | AA | M | 26 | 535 | 0.95 | 0.7 | DTG/ABC/3TC | AHI |
| DV-02 | W | M | 57 | 847 | 32 | 9.4 | EFV/TDF/FTC | CHI |
| DV-03 | W | M | 31 | 278 | 6.69 | 6.45 | TVD/ETR | CHI |
| DV-04 | AA | F | 37 | 873 | 16 | 12 | TVD/ATV | AHI |
| DV-05 | W | F | 55 | 893 | 6.7 | 6 | FTC/RPV/TAF | CHI |
| DV-06 | W | M | 60 | 902 | 7.28 | 6.84 | ATV/FTC/TDF/RTV | CHI |
| DV-07 | W | M | 23 | 668 | 1.95 | 1.86 | DTG/ABC/3TC | CHI |
| DV-08 | W/H | M | 28 | 1128 | 0.97 | 0.91 | DTG/ABC/3TC | AHI |
| DV-09 | AA | F | 46 | 835 | 6.9 | 6.6 | EFV/TDF/FTC | CHI |
| DV-10 | W | M | 50 | 574 | 4.04 | 4 | EVG/COBI/FTC/TDF | AHI |
| DV-11 | W | M | 47 | 516 | 2.33 | 2.26 | EVG/COBI/FTC/TDF | AHI |
| DV-12 | AA | M | 62 | 834 | 2.03 | 1.89 | DTG/ABC/3TC | CHI |
| **Mean** | **--** | **--** | **43.5** | **740.25** | **7.32** | **4.91** | **--** | **--** |

* (<50 copies HIV RNA/ml)
$^{\Psi}$Clinical characteristics at first donation

**Table S2:** Evolution of HIV p24 in wells in the DEVO assay. Wells are scored positive on day 8 if confirmed on day 12 (confirmed wells are shown in blue). Representative assay using resting CD4+ T cells from participant DV-09 is shown. High, above the upper limit of quantitation. Low, below the limit of detection.

| Cell Dilution (x $10^6$) | Well Number | HIV p24 Day 8 (fg/ml) | HIV p24 Day 12 (fg/ml) |
|---|---|---|---|
| 0.5 | A1 | **High** | **High** |
| 0.5 | A2 | **313.85** | **3285.80** |
| 0.5 | A3 | 1.75 | 5.79 |
| 0.5 | A4 | 33.16 | 5.37 |
| 0.5 | A5 | 0.76 | 0.05 |
| 0.5 | A6 | 3.10 | 6.19 |
| 0.5 | A7 | **2694.202** | **2722.05** |
| 0.5 | A8 | 34.14 | 9.04 |
| 0.5 | A9 | Low | Low |
| 0.5 | A10 | 6.87 | 7515.01 |
| 0.5 | A11 | 37.88 | 6935.88 |
| 0.5 | A12 | **11671.77** | **13328.83** |
| 0.1 | B1 | 0.91 | 57.00 |
| 0.1 | B2 | **4000.04** | **High** |
| 0.1 | B3 | 3.14 | 3.23 |
| 0.1 | B4 | 16.37 | 1.42 |
| 0.1 | B5 | Low | 0.96 |
| 0.1 | B6 | 0.59 | 7633.96 |
| 0.1 | B7 | 1.95 | Low |
| 0.1 | B8 | Low | 1.40 |
| 0.1 | B9 | 0.98 | Low |
| 0.1 | B10 | 3.28 | 12.29 |
| 0.1 | B11 | 2.82 | 5.38 |
| 0.1 | B12 | Low | 14291.94 |
| 0.05 | C1 | 1.78 | 79.11 |
| 0.05 | C2 | 1.88 | 18.41 |
| 0.05 | C3 | 3.10 | 6.61 |
| 0.05 | C4 | 8.50 | 231.36 |
| 0.05 | C5 | Low | 1.80 |
| 0.05 | C6 | 3.74 | 3.04 |
| 0.05 | C7 | Low | 1.37 |
| 0.05 | C8 | Low | 1.77 |
| 0.05 | C9 | 0.20 | 0.19 |
| 0.05 | C10 | 5.42 | Low |
| 0.05 | C11 | Low | 2.71 |
| 0.05 | C12 | Low | 16.25 |
| 0.025 | D1 | 7.526 | 0.54 |
| 0.025 | D2 | 1.724 | 35.01 |
| 0.025 | D3 | **High** | **High** |
| 0.025 | D4 | 6.99 | 6.12 |
| 0.025 | D5 | 5.03 | 1.54 |
| 0.025 | D6 | 15.88 | 60.97 |
| 0.025 | D7 | 1.80 | 1.25 |
| 0.025 | D8 | 2.83 | 2.57 |
| 0.025 | D9 | 2.39 | 3529.38 |
| 0.025 | D10 | 1.45 | 3.42 |
| 0.025 | D11 | 0.07 | 4.84 |
| 0.025 | D12 | 1.84 | 4.20 |

**Table S3:** Evolution of HIV p24 in wells in the DEVO assay. Wells are scored positive on day 8 if confirmed on day 12 (confirmed wells are shown in blue). Representative assay using resting CD4+ T cells from participant DV-02 is shown. High, above the upper limit of quantitation. Low, below the limit of detection.

| Cell Dilution (x $10^6$) | Well Number | p24 Day 8 (fg/ml) | HIV p24 Day 12 (fg/ml) |
|---|---|---|---|
| 0.5 | A1 | 1.45 | Low |
| 0.5 | A2 | **443.00** | **347.24** |
| 0.5 | A3 | 1.64 | 248.54 |
| 0.5 | A4 | Low | 41.04 |
| 0.5 | A5 | **131.66** | **133.06** |
| 0.5 | A6 | 237.58 | 85.85 |
| 0.5 | A7 | Low | 33.29 |
| 0.5 | A8 | Low | 19.61 |
| 0.5 | A9 | Low | 24.37 |
| 0.5 | A10 | Low | 49.26 |
| 0.5 | A11 | Low | 20.84 |
| 0.5 | A12 | **1471.49** | **129519.94** |
| 0.25 | B1 | Low | Low |
| 0.25 | B2 | **34537.62** | **High** |
| 0.25 | B3 | Low | High |
| 0.25 | B4 | Low | Low |
| 0.25 | B5 | 50.91 | 25.49 |
| 0.25 | B6 | **3047.45** | **High** |
| 0.25 | B7 | **307.82** | **279.21** |
| 0.25 | B8 | Low | 20.55 |
| 0.25 | B9 | 1.73 | 15.56 |
| 0.25 | B10 | 1.91 | 43.95 |
| 0.25 | B11 | **16324.21** | **High** |
| 0.25 | B12 | Low | 13.12 |
| 0.1 | C1 | 1.50 | Low |
| 0.1 | C2 | 0.41 | Low |
| 0.1 | C3 | **4480.21** | **1872.05** |
| 0.1 | C4 | 8.85 | 2.07 |
| 0.1 | C5 | 309.07 | 163.33 |
| 0.1 | C6 | Low | 30.61 |
| 0.1 | C7 | **58843.07** | **High** |
| 0.1 | C8 | 0.01 | 16.11 |
| 0.1 | C9 | Low | 22.22 |
| 0.1 | C10 | Low | 967.62 |
| 0.1 | C11 | 2.02 | 22.93 |
| 0.1 | C12 | Low | 19.25 |
| 0.05 | D1 | Low | Low |
| 0.05 | D2 | 4.13 | Low |
| 0.05 | D3 | **106.98** | **235.21** |
| 0.05 | D4 | Low | Low |
| 0.05 | D5 | 38.61 | 26.43 |
| 0.05 | D6 | 0.14 | 18.65 |
| 0.05 | D7 | Low | 421.03 |
| 0.05 | D8 | Low | 21.48 |
| 0.05 | D9 | Low | 18.94 |
| 0.05 | D10 | Low | 17.72 |
| 0.05 | D11 | Low | 17.37 |
| 0.05 | D12 | Low | 26.13 |
| 0.025 | E1 | Low | Low |
| 0.025 | E2 | Low | Low |
| 0.025 | E3 | Low | Low |
| 0.025 | E4 | **5267.67** | **41626.69** |
| 0.025 | E5 | **85.82** | **218.06** |
| 0.025 | E6 | Low | 32.45 |
| 0.025 | E7 | Low | 23.47 |
| 0.025 | E8 | 0.56 | 14.45 |
| 0.025 | E9 | Low | 27.67 |
| 0.025 | E10 | Low | 16.49 |
| 0.025 | E11 | Low | 19.60 |
| 0.025 | E12 | Low | 21.52 |
